# Supplementary material for: The microbiome biomarkers of pregnant women’s vaginal area predict preterm prelabor rupture in Western China
Source: Front Cell Infect Microbiol. 2024 Oct 31;14:1471027. doi: 10.3389/fcimb.2024.1471027 (PMC11560878; doi:10.3389/fcimb.2024.1471027)
Supplement: Supplementary file 1 [file DataSheet1.zip › compare_1/Community/Pieplot/genus/sample/C19.genus.pieplot.pdf]

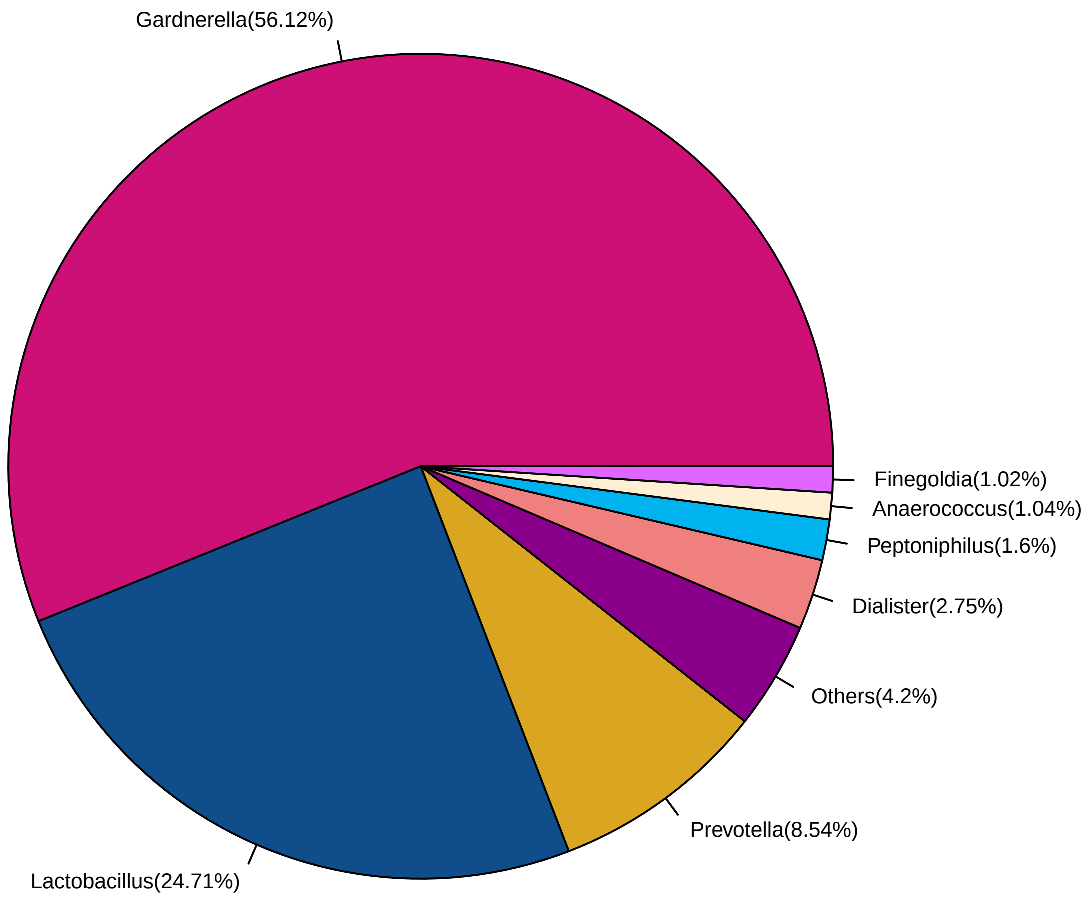
Gardnerella(56.12%)
Finegoldia(1.02%)
Anaerococcus(1.04%)
Peptoniphilus(1.6%)
Dialister(2.75%)
Others(4.2%)
Prevotella(8.54%)
Lactobacillus(24.71%)
